# Meta-analysis of neuron specific enolase in predicting pediatric brain injury outcomes

**DOI:** 10.17179/excli2017-405

**Published:** 2017-07-03

**Authors:** Babak Nakhjavan-Shahraki, Mahmoud Yousefifard, Alireza Oraii, Arash Sarveazad, Mostafa Hosseini

**Affiliations:** 1 Sina Trauma and Surgery Research Center, Tehran University of Medical Sciences, Tehran, Iran; 2 Physiology Research Center and Department of Physiology, Faculty of Medicine, Iran University of Medical Sciences, Tehran, Iran; 3 Department of Medicine, School of Medicine, Tehran University of Medical Sciences, Tehran, Iran; 4 Colorectal Research Center, Iran University of Medical Sciences, Tehran, Iran; 5 Department of Epidemiology and Biostatistics, School of Public Health, Tehran University of Medical Sciences, Tehran, Iran

**Keywords:** biomarker, pediatric, traumatic brain injury

## Abstract

A reliable biomarker has not been identified to predict the outcome of traumatic brain injury (TBI) in children. Therefore, the present systematic review and meta-analysis aimed to assess the association between neuron specific enolase (NSE) and traumatic brain injury (TBI) in children. Two independent reviewers searched electronic databases of EMBASE, Cochrane library, Medline and Scopus and then they summarized the results and did a quality control check. At the end, standardized mean difference (SMD) with 95 % confidence interval (CI) and performance of NSE were assessed. 10 studies were included in the present meta-analysis. Average serum (SMD=1.3; 95 % CI: 0.5 to 2.1; p=0.001) and CSF levels (SMD=2.45; 95 % CI: 1.04 to 3.8; p<0.0001) of NSE biomarker were significantly higher in children with TBI with unfavorable outcome compared with other children. Serum NSE had an area under the curve, sensitivity and specificity of 0.75 (95 % CI: 0.72 to 0.79), 0.74 (95 % CI: 0.64 to 0.82) and 0.69 (95 % CI: 0.59 to 0.77), respectively in prediction outcome of TBI. Positive likelihood ratio, negative likelihood ratio and diagnostic odds ratio of serum NSE were 2.4 (95 % CI: 1.7 to 3.3), 0.38 (95 % CI: 0.26 to 0.55) and 6.0 (95 % CI: 3.0 to 12.0), respectively. The results show that the performance of NSE is in a moderate level in prediction of unfavorable outcome in children with TBI. However, data in this aspect is not sufficient and more studies are needed.

## Introduction

Traumatic brain injuries (TBI) comprise 2.8 million emergency department visits, hospitalizations, and deaths annually. These amounts are even more than annual incidence of other neurodegenerative disorders combined (Taylor et al., 2017[42]). TBI is the most important cause of mortality and morbidity among children and these morbidities consist of a wide spectrum from transient to persistent injuries (Thurman, 2016[43]). Studies show that early identification of high risk patients with TBI leads to reduction in disease burden, mortality and morbidity of these injuries. However, reliable criteria have not been developed yet for prediction of the presence of brain lesions in patients with head trauma, especially children (Dayan et al., 2017[7]; DeFazio et al., 2014[8]). Therefore, researchers are searching for an ideal marker which has high accuracy and validity for prediction of brain injury, rises only after brain injury, rises rapidly in serum and has a time association with the beginning of trauma (Wilkinson et al., 2016[46]). The presence of such a marker gives physicians an opportunity to follow patients with head trauma without neurologic signs more accurately.

There is a cascade of molecular, cellular and biochemical changes after TBI which worsens traumatic brain injury in children. Therefore, the severity of head injury is more accurately assessed by following molecules, peptides and biomarkers released as result of these changes. Neuron specific enolase (NSE) is one of these neuropeptides (Rodríguez-Rodríguez et al., 2016[36]). NSE is a 75 kilodalton gama homodimer specific for neurons and neuroendocrine tissue (Haque et al., 2016[15]; Shi et al., 2017[38]). Its level is not considerable in other tissue. As this protein is specific to neural tissue, its serum or CSF level more commonly rises in case of neural tissue injury or disease. Its serum and CSF level rises in early hours after trauma and therefore its measurement can be helpful in identifying TBI. In the past decade, NSE was primarily considered as a peripheral biomarker of permeability of the blood brain barrier. For example, Rodríguez-Rodrígue et al. (2016[36]) showed elevated levels of this protein indicate the presence of a TBI. Papa et al. (2015[34]) showed that elevated level of NSE in traumatic patients is a possible screening tool for diagnosing inflicted TBI. Therefore, using this marker may be an accurate and sensitive tool for assessing brain injury in a molecular level before occurrence of extensive injuries. A meta-analysis by Cheng et al. (2014[4]) showed that serum level of this protein rises significantly in adult patients with moderate to severe traumatic injuries and can be used as a tool for assessing the severity of brain injury. Mercier et al. (2016[30]) reached similar results. However, using NSE for identification of traumatic brain injury in children is controversial. A consensus has not been reached yet in the field of pediatrics. Therefore, the present systematic review and meta-analysis aimed to assess the association between NSE and traumatic brain injury in children.

## Materials and Methods

### Search strategy

The present study was designed based on instructions on Meta-analysis of Observational Studies in Epidemiology (MOOSE) statement (Stroup et al., 2000[41]). Two independent reviewers searched electronic databases of EMBASE, Cochrane library, Medline and Scopus without a time limit. The search strategy was based on words related to traumatic brain injury and protein biomarkers emphasizing on NSE protein. Keywords were obtained using MeSH (Medical Subject Headings) in PubMed database and Emtree in Embase database. Additionally, in order to find additional articles and unprinted data, a hand-search was done in the bibliography of relevant studies, Google Scholar, Google motor engine, ProQuest and Trip database. Keywords used in the search are shown in Table 1(Tab. 1).

### Selection criteria

In the present study, observational studies on assessing NSE protein level in children (under 18) with TBI were entered. Inclusion criteria were identification of traumatic brain injury with well-validated diagnostic criteria, measurement of serum NSE level during 24 hours after trauma, assessment of the outcome of brain injury based on CT scan, Magnetic resonance imaging (MRI) or Glasgow outcome scale (GOS) (McMillan et al., 2016[28]), consisting of a group with favorable and a group with unfavorable outcome, reporting mean serum NSE level and its standard deviation (derived from the article or contacting the authors) or performance characteristics of NSE in prediction of TBI related outcome. Animal studies and studies lacking two groups of patients with and without lesions were excluded from the study. All retrospective and prospective studies were included in the study.

### Quality assessment and data extraction

The method of data extraction has been precisely reported in previous studies done by authors of the present study (Ebrahimi et al., 2014[9]; Ghelichkhani et al., 2016[14]; Hassanzadeh‐Rad et al., 2016[16]; Hosseini et al., 2015[19][20], 2016[21]; Izadi et al., 2016[24][25]; Nakhjavan-Shahraki et al., 2017[33]; Rahimi-Movaghar et al., 2016[35]; Safari et al., 2016[37]; Yousefifard et al., 2016[47][48][49][50]). In summary, search results were combined and same references were excluded using EndNote (version X5, Thomson Reuters, 2011). Title and summary of extracted articles were assessed by two independent researchers and were entered in a predesigned form. Results of the systematic search of the present study were depicted by a flow chart which was designed based on PRISMA statement instructions (Moher et al., 2009[32]). Extracted data consisted of data regarding study design, patient characteristics (age, sex, severity of trauma), method of measuring NSE level, its storage temperature, assessed outcomes, sample size of the studies, mean, standard deviation, sensitivity, specificity of NSE in identification of brain lesions, final diagnosis of patients with traumatic brain injury and cut off point of NSE level. In case of inaccessibility to authors, estimation methods were used to calculate mean and standard deviation from median and range of data (Higgins and Green, 2011[18]; Hozo et al., 2005[22]). If results were reported in charts, data were extracted from these charts using the method introduced by Sistrom and Mergo (2000)[40].

Quality of studies was assessed using suggested instructions in quality assessment of studies of diagnostic accuracy included in systematic reviews version 2 (QUADAS-2) (Whiting et al., 2011[45]).

### Statistical analysis

Data analysis was done using STATA version 11.0 (Stata Corporation, College Station, TX). Patients were categorized into two groups of good outcome (Full recovery or mild disability) and unfavorable outcome (moderate to severe disability and death). All studies were categorized and summarized based on mean value and standard deviation. As studies had used different methods for measuring NSE level, standardized mean difference (SMD) was used in the analyses as effect size using Hedges' g calculations. Heterogeneity between studies was assessed using I^2 ^test with I^2^ more than 75 percent or a p value of less than 0.1 (indicating heterogeneity between studies). The meta-analysis was done using fixed effect model if studies were homogenous, otherwise a random effect model was used. In the present study, subgroup and sensitivity analyses were done in order to reduce heterogeneity. Funnel plot and Egger's test were used in order to identify publication bias (Egger et al., 1997[10]). In addition, summary receiver operator characteristic (SROC) curve, sensitivity, specificity, positive and negative likelihood ratio and diagnostic odds ratio of neuron specific enolase in prediction of TBI related outcome were calculated to assess the performance of NSE.

## Results

### Characteristic of included studies

**Figure 1:**
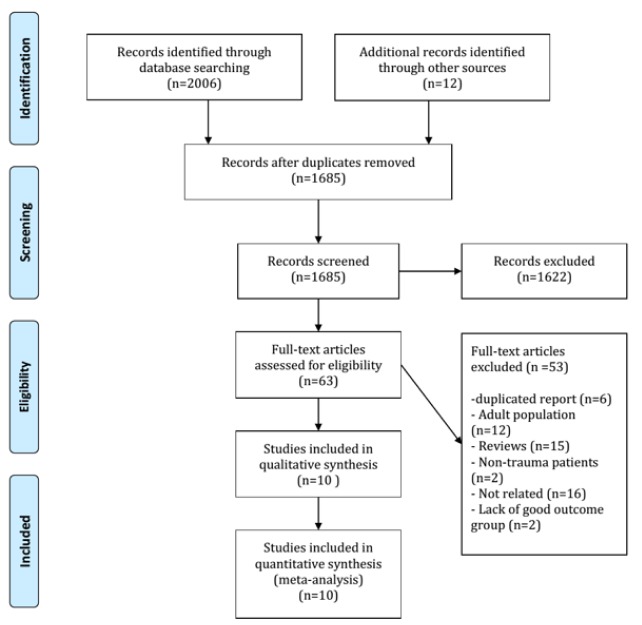
Flowchart of included studies

Primary search in databases resulted in finding of 2006 articles of which repetitive articles were omitted resulting in a total of 1685 studies. After primary screening, full texts of 63 articles were read and 10 studies were included in the meta-analysis (Bandyopadhyay et al., 2005[1]; Berger et al., 2002[3], 2005[2]; Chiaretti et al., 2009[5]; Fridriksson et al., 2000[12]; Geyer et al., 2009[13]; Lo et al., 2010[27]; Shore et al., 2007[39]; Varma et al., 2003[44]; Žurek and Fedora, 2012[51]). A flow chart of included studies is depicted in Figure 1(Fig. 1). These studies contained 721 children (mean age of 6.9+1.9, 58.5 percent boys). According to definitions in studies, 343 children (48.2 %) were in the group of good outcome and 373 children (52.5 %) were in the group of unfavorable outcome. In one study (Žurek and Fedora, 2012[51]), the value of NSE level in prediction of two outcomes (mortality and poor neurological outcome) was assessed. Therefore, two separate experiments were extracted from the mentioned study. At the end, data from 11 experiments were entered in the meta-analysis. 7 experiments (63.6 %) had assessed serum NSE level (Bandyopadhyay et al., 2005[1]; Berger et al., 2005[2]; Chiaretti et al., 2009[5]; Fridriksson et al., 2000[12]; Geyer et al., 2009[13]; Lo et al., 2010[27]; Žurek and Fedora, 2012[51]) while 4 experiments (36.4 %) had assessed CSF level of this biomarker (Berger et al., 2002[3]; Chiaretti et al., 2009[5]; Shore et al., 2007[39]; Varma et al., 2003[44]). Characteristics of included studies are shown in Table 2(Tab. 2) (References in Table 2: Bandyopadhyay et al., 2005[1]; Berger et al., 2002[3]; Berger et al., 2005[2]; Chiaretti et al., 2009[5]; Fridriksson et al., 2000[12]; Geyer et al., 2009[13]; Lo et al., 2010[27]; Shore et al., 2007[39]; Varma et al., 2003[44]; Zurek and Fedora, 2012[51]).

### Quality control

**Figure 2:**
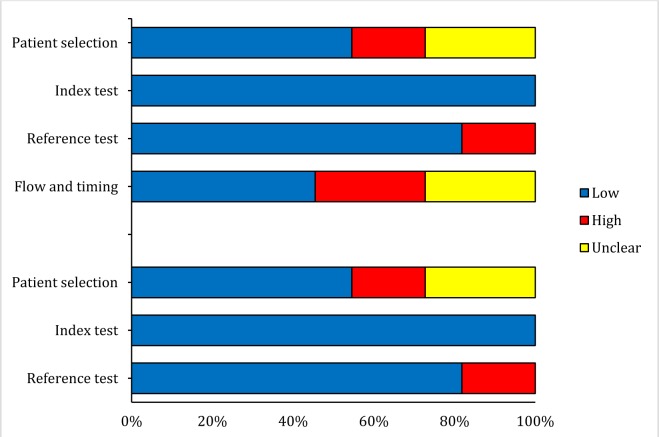
Risk of bias and applicability concerns of included studies assessing value of neuron specific enolase concentrations in prognosis in children with traumatic brain injury

Studies were controlled based on their methodology and they were scored using instructions of QUADAS-2. Details of quality control of included studies are depicted in Figure 2(Fig. 2). The assessment of outcome was done blindly in only 4 studies.

### Meta-analysis

#### The value of serum NSE level in prediction of outcome of TBI in children

6 studies including 7 experiments were entered in order to assess the value of serum NSE level in prediction of outcome of TBI in children (Bandyopadhyay et al., 2005[1]; Berger et al., 2005[2]; Fridriksson et al., 2000[12]; Geyer et al., 2009[13]; Lo et al., 2010[27]; Žurek and Fedora, 2012[51]). 

**Figure 3:**
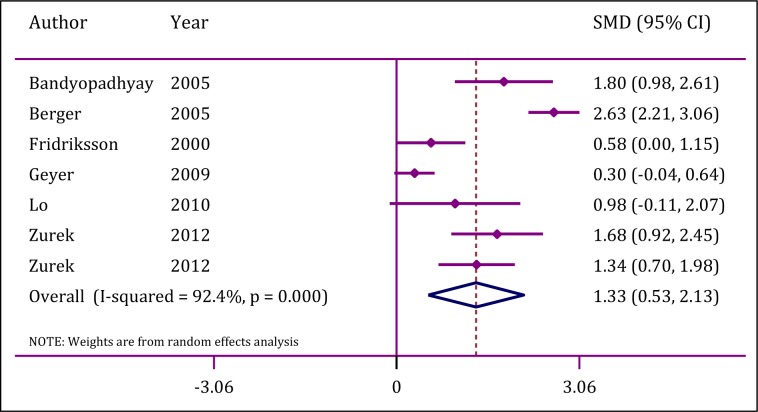
Forrest plot of serum neuron specific enolase in prediction of traumatic brain injury in children. CI: Confidence interval; SMD: Standardized mean differences

Analyses showed a significant heterogeneity between studies (I^2^ = 92.4; p<0.0001). However, publication bias was not observed (p=0.62). Mean serum NSE level in children with TBI related unfavorable outcome was significantly higher than levels observed in other children (SMD=1.3; 95 % CI: 0.5 to 2.1; p=0.001) (Figure 3(Fig. 3); References in Figure 3: Bandyopadhyay et al., 2005[1]; Berger et al., 2005[2]; Fridriksson et al., 2000[12]; Geyer et al., 2009[13]; Lo et al., 2010[27]; Zurek and Fedora, 2012[51]).

The source of heterogeneity was sought using subgroup analysis. Meta-regression showed that differences in assessed outcome (OR=3.4; 95 % CI: 1.04-12.6; p=0.03), blinding status (OR=77.3; 95 % CI: 11.6-515.2; p=0.002) and patient selection method (OR=7.5; 95 % CI: 1.8-31.7; p=0.02) were the most important source of heterogeneity (Table 3(Tab. 3)).

#### The value of CSF level of NSE in prediction of the outcome of TBI in children

**Figure 4:**
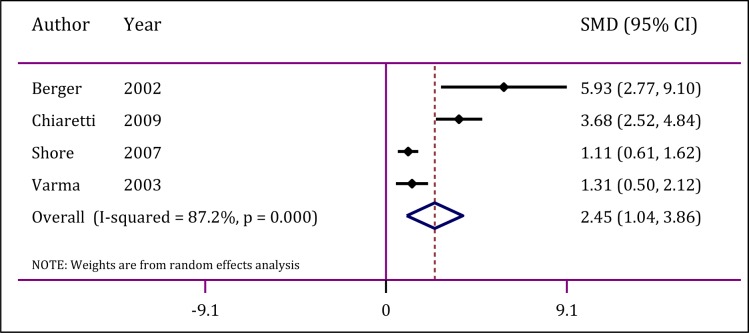
Forrest plot of cerebrospinal fluid neuron specific enolase in prediction of traumatic brain injury in children. CI: Confidence interval; SMD: Standardized mean differences

In literature review, only 4 studies had assessed the value of CSF level of NSE in predicting the outcome of TBI in children (Berger et al., 2002[3]; Chiaretti et al., 2009[5]; Shore et al., 2007[39]; Varma et al., 2003[44]). A significant heterogeneity was also observed among these studies (I^2 ^=87.2 %; p<0.001). Publication bias was not observed (p=0.12). The findings show that mean CSF level of NSE in children with TBI related unfavorable outcome is significantly higher than levels observed in children with good outcome (SMD=2.45; 95 % CI: 1.04 to 3.8; p<0.0001) (Figure 4(Fig. 4); References in Figure 4: Berger et al., 2002[3]; Chiaretti et al., 2009[5]; Shore et al., 2007[39]; Varma et al., 2003[44]). A subgroup analysis could not be done due to the small number of studies in this section.

#### Performance of NSE in prediction of pediatric TBI

**Figure 5:**
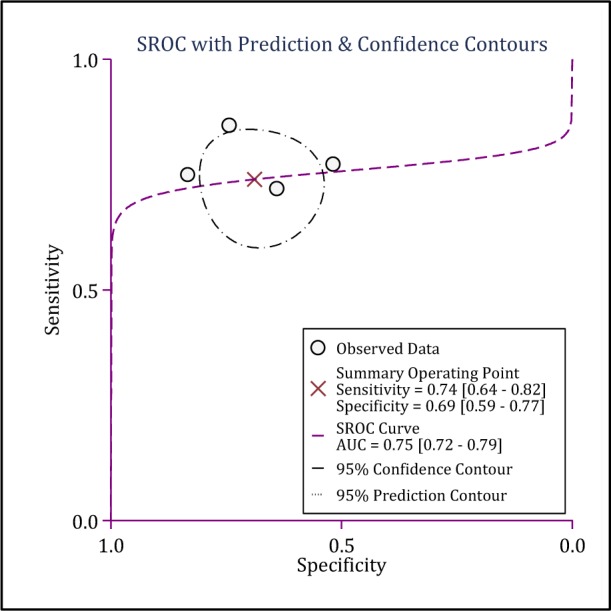
Summary receiver operator characteristic (SROC) curve of neuron specific enolase in prediction of pediatric traumatic brain injury. AUC: Area under the curve

Screening performance characteristics of NSE in prediction of pediatric TBI related outcome was done in 4 studies (Bandyopadhyay et al., 2005[1]; Fridriksson et al., 2000[12]; Geyer et al., 2009[13]; Žurek and Fedora, 2012[51]). All these studies focused on serum levels of NSE. Cut offs of NSE were varied between 11.36 to 25.5 ng/ml (Table 2(Tab. 2)). SROC, sensitivity and specificity of serum NSE level in prediction of TBI related outcome were 0.75 (95 % CI: 0.72 to 0.79), 0.74 (95 % CI: 0.64 to 0.82) and 0.69 (95 % CI: 0.59 to 0.77), respectively. Positive likelihood ratio, negative likelihood ratio and diagnostic odds ratio of serum NSE level were 2.4 (95 % CI: 1.7 to 3.3), 0.38 (95 % CI: 0.26 to 0.55) and 6.0 (95 % CI: 3.0 to 12.0), respectively (Figure 5(Fig. 5)).

## Discussion

The present meta-analysis assessed the diagnostic value of NSE in children with TBI. The findings show that both serum and CSF levels of NSE are higher in children with unfavorable outcome. However, area under the curve of NSE indicates moderate performance of this biomarker in prediction of outcome in children with TBI.

In comparison with other studies, Daoud et al. (2014[6]) reported a strong association between NSE level and unfavorable outcome in children with TBI in a systematic review in 2013 consisting of 3 studies (Daoud et al., 2014[6]). Kochanek et al. (2013[26]) also reported similar findings in a narrative review. In addition, Menascu et al. (2010[29]) considered NSE as a probable biomarker of prediction of outcome in children with TBI. In a meta-analysis by Cheng et al. (2014[4]) NSE level had direct relationship with mortality and unfavorable outcome in adults with TBI. However, discriminatory power of NSE in prediction of mortality and neurologic outcome was moderate. Mercier et al. (2012[31]) showed that there is a meaningful relationship between NSE level and outcome in adults with TBI although, optimal clinical threshold of this biomarker in prediction of outcome of TBI has not been identified yet. Finding of these two meta-analyses are consistent with findings of the present study.

The present study showed that both serum and CSF level of NSE have direct relationship with unfavorable outcome in children with TBI. However, there was a significant heterogeneity among studies. Differences in assessed outcome, blinding status and patient selection method were the most important sources of heterogeneity in assessing the value of serum NSE level. Performance of serum NSE level in prediction of unfavorable outcome was in a moderate level. This can be due to the fact that brain is not the only source of NSE and it can also be found in platelets and red blood cells (Elson and Ward, 1994[11]). In addition, trauma to other organs can lead to elevations in serum levels of this biomarker. Therefore CSF level of NSE could be a more accurate predictor of severity of brain injury. However, there is no study assessing the performance characteristics of CSF concentration of NSE in prediction of outcome in children with TBI. In addition, a lumbar puncture is needed in order to obtain CSF level of NSE and such invasive procedures come with ethical limitations in all children with TBI.

Subgroup analysis showed that NSE has different values in predicting TBI, depending on the outcome under assessment. The reason of this finding might be the severity of injury. Glasgow outcome scale (GOS) is a scale consisting of six categories of good recovery, mild disability, moderate disability, severe disability, persistent vegetative state and death (McMillan et al., 2016[28]). Most studies have divided GOS to two groups of poor outcome (severe disability, persistent vegetative state and death) and good outcome (good recovery, mild disability and moderate disability) and then have assessed the value of NSE in predicting the mentioned outcomes. It seems that NSE level rises dramatically in poor outcome patients and subsequently this increases the predictive value of NSE.

Blinding status of the observer is another influential factor on the value of NSE. Studies analyzing data in a blind manner have reported greater values for NSE. This indicates the importance of blinding status of observers in designing the methodology in order to get more accurate and more reliable results (Hróbjartsson et al., 2014[23]). 

In addition, different sampling methods alter the results regarding the value of NSE. Studies using convenience sampling have reported a greater value for NSE in prediction of the outcome of TBI in children. This might be due to the possible selection bias present in the convenience sampling (Hedt and Pagano, 2011[17]). However, NSE was capable of predicting the outcome of TBI when consecutive sampling was used. Hence, selection bias did not have a significant effect on final interpretation of the results in the present meta-analysis.

Although an extensive search was done in databases to find maximum number of related articles, only 10 studies were included in the present meta-analysis with most recent one published in 2012. In order to find more recent articles a hand-search was done in Google Scholar, Google motor engine, Trip database, ProQuest database and bibliography of relevant studies. Only one study in 2016 was found (Wilkinson et al., 2016[46]) in which needed data were not presented in the article. Corresponding author and other authors of the mentioned study were contacted by email in order to get the needed data however; there was no response after two email contacts. In general, it seems that there is less attention to the value of NSE level in children with TBI although, there is still a wide gap in this field of study. Inability to find an optimal cut point for NSE was one of the limitations of the present study. Additionally, small number of studies assessing CSF level of this biomarker lead to a significant heterogeneity among included studies that made the source of heterogeneity less obvious.

## Conclusion

Finding a reliable biomarker in prediction of the outcome of TBI in children can be useful in management of these patients. In the present study, the value of NSE level in prediction of the outcome of TBI in children was assessed in a meta-analytical approach. The findings indicate that the performance of NSE level in prediction of unfavorable outcome in children with TBI is in a moderate level. However, lack of sufficient number of studies is felt in this aspect and further research is need.

## Acknowledgement

None.

## Fund

None.

## Conflict of interest

The authors declared no conflict of interest.

## Author contribution

- Study design and conception: Mostafa Hosseini, Mahmoud Yousefifard, Babak Nakhjavan-Shahraki, Arash Sarveazad
- Data gathering: Babak Nakhjavan-Shahraki, Mahmoud Yousefifard, Alireza Oraii
- Analysis: Mostafa Hosseini
- Writing the first draft: Mahmoud Yousefifard, Alireza Oraii

## Figures and Tables

**Table 1:**
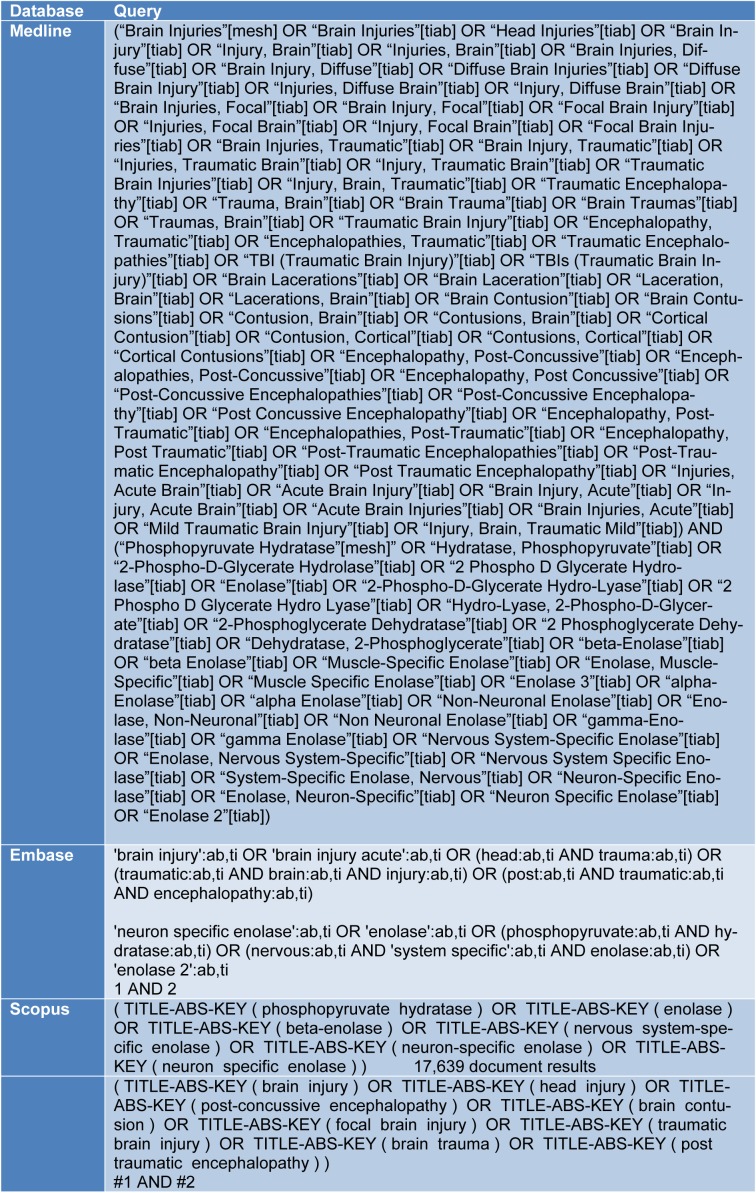
Search queries in Medline, Embase and Scopus databases

**Table 2:**
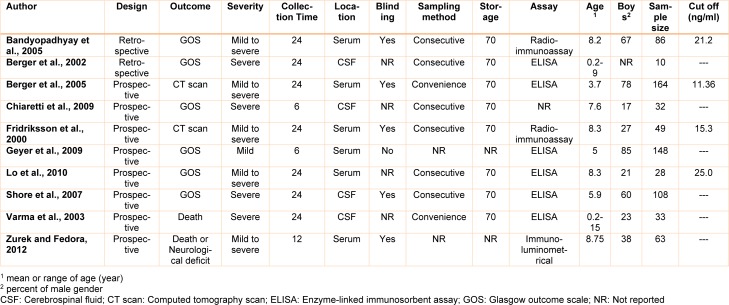
Summery of included studies' characteristics

**Table 3:**
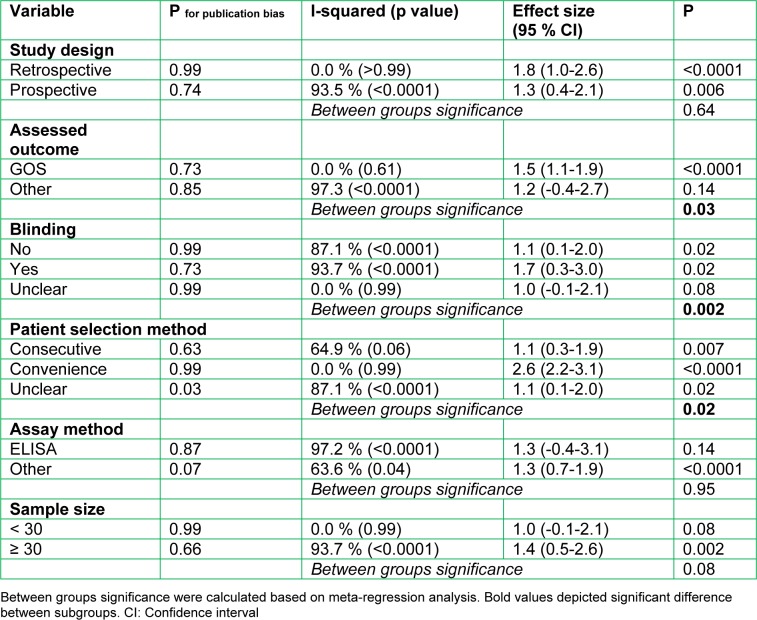
Subgroup analysis of value of serum neuron specific enolase in prediction of pediatric traumatic brain injury
